# Oxidized Cell-Free DNA Rapidly Skews the Transcriptional Profile of Brain Cells toward Boosting Neurogenesis and Neuroplasticity

**DOI:** 10.3390/cimb43030112

**Published:** 2021-10-13

**Authors:** Anton D. Filev, Svetlana V. Kostyuk, Pavel E. Umriukhin, Vladimir M. Pisarev

**Affiliations:** 1 Research Centre for Medical Genetics (RCMG), 115478 Moscow, Russia; svet-vk@yandex.ru (S.V.K.); pavelum@mail.ru (P.E.U.); vpisarev@gmail.com (V.M.P.); 2 Federal Research and Clinical Center of Intensive Care Medicine and Rehabilitology, V.A. Negovsky Research Institute of General Reanimatology, 107031 Moscow, Russia; 3 Department of Normal Physiology, I.M. Sechenov First Moscow State Medical University (Sechenov University), 119991 Moscow, Russia

**Keywords:** neurogenesis, inflammation, oxidized cell-free DNA, mRNA

## Abstract

Cell-free DNA (cfDNA) is liberated and accumulated in neural tissue due to cell damage. The oxidative and nitrosative stress in the brain that accompanies various pathological conditions has been shown to increase the oxidation of cellular and cell-free DNA. Whether the high concentration of non-oxidized and oxidized cfDNA may affect the transcriptome response of brain cells has not been studied. In the current work, we studied whether cfDNA fragments affect several key pathways, including neurogenesis, at the level of gene expression in brain cells. In the study, primary rat cerebellum cell cultures were used to assess the effects of oxidized and non-oxidized cfDNA on the expression of 91 genes in brain cells. We found that only oxidized cfDNA, not non-oxidized cfDNA, significantly altered the transcription in brain cells in 3 h. The pattern of change included all 10 upregulated genes (*S100A8*, *S100A9*, *S100b*, *TrkB*, *Ngf*, *Pink1*, *Aqp4*, *Nmdar*, *Kcnk2*, *Mapk1*) belonging to genes associated with neurogenesis and neuroplasticity. The expression of inflammatory and apoptosis genes, which oppose neurogenesis, decreased. The results show that the oxidized form of cfDNA positively regulates early gene expression of neurogenesis and neuroplasticity. At the same time, the question of whether chronic elevation of cfDNA concentration alters brain cells remains unexplored.

## 1. Introduction

Every day, many thousands of neurons are born and differentiate from neural precursors, supporting brain tissue renewal [1,2]. An imbalance between death, birth, and differentiation may contribute to various neuropathologies [3,4]. It has been found that in chronic stress, neurodegenerative diseases, traumatic brain injury, sepsis, and stroke, the increased cell-free DNA (cfDNA) concentration in plasma is associated with the course and/or outcome [5,6,7,8,9]. Oxidative stress after cerebral ischemia is shown to induce DNA damage in the brain [10]. Our previous studies demonstrated that cfDNA in plasma might also contain oxidized nucleotides in several neurological diseases and may be associated with the disease course [11,12]. It is believed that mitochondrial DNA is less methylated [13] and more oxidized (with 10–15 times more 8-oxo-2′-deoxyguanosine (8-oxo-dG) residue [14]) than nuclear DNA. cfDNA molecules containing 8-oxo-dG demonstrate more prominent biological effects [15], presumably due to faster penetration into various cells (rat cerebellum cells, MCF7, fibroblasts) [15,16,17]. Released from cells mainly as part of exosomes [18], cfDNA is taken up by both the nearest bystander cells and distant outlying cells. Intracellularly, cfDNA molecules bind specific DNA sensors (TLR9, NLRP3, cGAS, etc.) to activate the inflammatory, oxidation, and adaptive responses. At the same time, cfDNA induces antioxidant mechanisms through increased levels of the *Nrf2* and *Hmox1* genes and protein expression [15,16,19].

While various effects of cfDNA on cells of different origin have been described, the mechanism of its action on brain cells remains unexplored. In the current study, we evaluated the early effects of oxidized and non-oxidized cfDNA on the expression of genes that control inflammation, oxidation, neurogenesis and neuroplasticity, apoptosis, autophagy/mitophagy, and DNA repair (a total of 91 genes).

## 2. Materials and Methods

### 2.1. Primary Cell Culture

Cerebellum tissue of 8-day-old Wistar rats was used for primary cell culture (PCC). Lysis solution (1 V 0.25% trypsin solution + 1 V Versene–EDTA solution) was added to the tissue and kept in a water bath for 15 min at 37 °C. Then the tube was centrifuged at 200× *g* for 30 s, and the supernatant was removed and washed with D-PBS solution and DMEM. The tissue was homogenized with a Pasteur pipette. The resulting cell suspension was passed through a 70 µm filter (SPL Life Sciences, Camarillo, CA, USA) on a 50 mL tube, then centrifuged at 200× *g* for 3 min, and the supernatant was removed. The cell pellet was diluted to the required volume with a neural cell culture medium (Neurobasal ™ Medium, B27 supplement, 2% FBS). All solutions and consumables were from PanEco (Moscow, Russia).

The cells were seeded on 6-well Nuclon ^TM^ plates (Thermo Fisher Scientific, Waltham, MA, USA) with pre-treated poly-D-lysine (Sigma-Aldrich, St. Louis, MO, USA) (concentration 50 μg/mL per hour, washed 3 times with deionized water, and dried in a sterile zone). The PCC was kept for 72 h in a CO_2_ incubator (5% CO_2_, 95% air, 37 °C, 98–100% humidity) (Eppendorf, Hamburg, Germany). The culture medium was changed every 3 days, and cell recovery was accessed by light microscopy (AxioVert, Carl Zeiss Microscopy, Jena, Germany).

### 2.2. CfDNA Purification and Oxidation

It is believed that in circulation, cfDNA accumulates in small fragments (150–180 b. p.) [20]. At the same time, DNA releases from cells to intracellular space of various sizes (200–10,000 b. p.) depending on the mechanisms of its production [21,22,23]. Previously, the effects of DNA fragments were studied using both long [17] and short [24] DNA fragments. For our experiments, we used total DNA from brain (cells and intracellular space) containing both genomic DNA and mitochondrial DNA. We pose that brain cells produce cfDNA of different sizes (200–10,000 b. p.), and those DNA fragments that we add to cell culture may mimic cfDNA liberating during brain cell damage.

DNA purification and oxidation were performed as described in [16,25]. Briefly, DNA was extracted from forebrain tissue of newborn rats using phenol-chloroform reagents. This method contains proteinase K and RNase treatments for protein and RNA removal, respectively. Then, DNA was oxidized by 3% hydrogen peroxide solution with UV λ = 312 (30 s). To ensure that the effect of oxidized cfDNA in cell culture is not due to presence of a residual hydrogen peroxide, the samples were treated as described in Table S2. Non-oxidized or oxidized cfDNA was added to the culture medium at a final concentration of 50 ng/mL. The average content of 8-oxo-dG was 400–600 per 10^6^ nucleotides.

### 2.3. Sampling and mRNA Purification

To analyze the gene expression, all procedures were performed in accordance with the manufacturers’ protocols for the reagent kits. Total RNA was isolated from brain tissue specimens using the RNeasy Mini Kit (Qiagen, Hilden, Germany) and treated with DNase I. The purity of the isolated RNA was determined spectrophotometrically by using a NanoDrop™ OneC (Thermo Fisher Scientific, Waltham, MA, USA). An RNA Clean & Concentrator–5 kit (Zymo Research, Irvine, CA, USA) was used to remove contaminants from the RNA samples. The RNA concentration was assessed using a Qubit 3.0 fluorometer (Thermo Fisher Scientific, Waltham, MA, USA). The RNA integrity number (RIN) was determined electrophoretically using an Agilent 2100 bioanalyzer and Agilent RNA 6000 Nano Kit (both from Agilent Technologies, Santa Clara, CA, USA); only samples with RIN >5 were used for further analysis.

### 2.4. RT PCR

RT PCR was performed with specific primers (Evrogen, Moscow, Russia) (Table 1) and SybrGreen intercalating dye (Invitrogen, Waltham, MA, USA) on a StepOnePlus™ Real-Time PCR System (Applied Biosystems, Waltham, MA, USA). The *Ppia* gene was chosen as the housekeeping gene.

### 2.5. Multiplex Gene Expression Analysis

To explore the expression of 91 genes in the brain tissue specimens by a previously created gene panel [26], the nCounter FLEX Analysis System and the CodeSet reagent kit (Nanostring Technology, Inc., Seattle, WA, USA) were employed. This technology allows direct multiplex assay of gene transcription activity. The method is based on detecting targets labelled with unique color barcodes attached to target-specific probes. The analysis was performed according to the manufacturer’s protocol. We selected 5 HKGs, *B2m*, *Gapdh*, *Hmbs*, *Ppia*, and *Ywhaz*, which have been used in various studies as proven HKGs [27,28,29]. We studied the gene expression for signaling pathways associated with inflammation, oxidation, autophagy, mitophagy, apoptosis, DNA repair, neurogenesis, and neuroplasticity. The expression level of the studied genes was normalized to the reference genes and then to the control group (without cfDNA addition).

### 2.6. Statistics

Analysis of variance (one-way ANOVA, the Holm–Sidak method), with a Bonferroni correction for multiple comparisons (*p* _adjusted_ = *p* _unadjusted_ * 91), was performed using SigmaStat 3.1 (Systat Software Inc., San Jose, CA, USA). Two-fold differences in expression vs. control were considered significant at *p* _adj_ < 0.01 for multiplex gene expression analysis, and *p* < 0.01 for RT PCR.

## 3. Results

### 3.1. Early Effects of Oxidized and Non-Oxidized cfDNA on Gene Expression in Brain Cells

**Figure 1:**
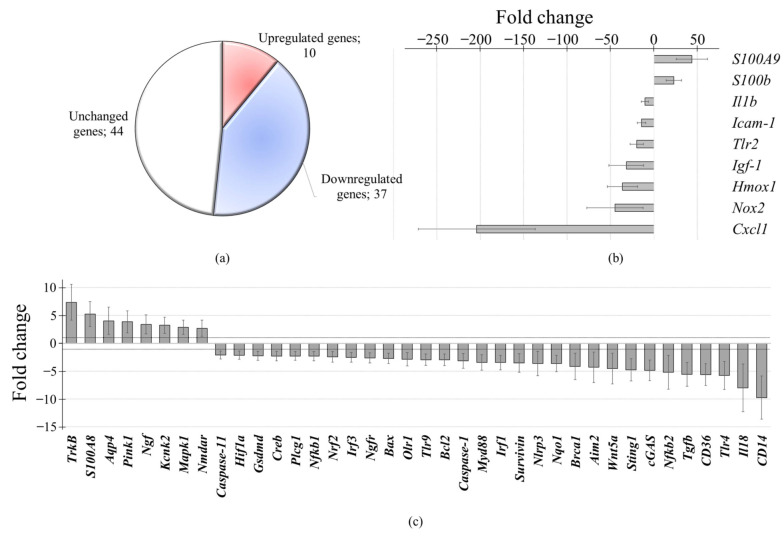
Multiplex gene expression analysis of cells of rat cerebellum after oxidized cell-free DNA treatment for 3 h. (**a**) Gene distribution according to type of change in gene expression. (**b**) High (more than 10-fold) alteration of gene expression. (**c**) Moderate (2- to 10-fold) alteration of gene expression. One-way ANOVA, Holm–Sidak method. *p* _adj_ < 0.0001 (all shown genes).

Multiplex gene expression analysis was used to assess the expression of 91 genes following 3 h incubation of rat brain cell cultures with oxidized and non-oxidized cfDNA. Non-oxidized cfDNA did not show any significant effect on the expression of the studied genes (Table S1). In contrast, oxidized cfDNA differentially changed the expression of 47 out of 91 genes (Figure 1).

Ten genes showed significantly increased expression profiles: S100 family (*S100b* 23-fold, *S100A8* 5.2-fold, *S100A9* 43.8-fold), neurogenesis and neuroplasticity (*TrkB* 7.4-fold, *Ngf* 3.4-fold, *Mapk1* 2.88-fold), mitophagy (*Pink1* 3.9-fold), *Nmdar* (2.69-fold), *Kcnk2* (3.24-fold), and *Aqp4* (4.02-fold) (Figure 1b,c and Table S1).

Expression of 37 genes decreased in the range of >10-fold (7 genes), 5- to 10-fold (6 genes), and 2- to 5-fold (24 genes) (Figure 1b,c and Table S1). The oxidized DNA-induced transcriptional profile included decreased expression of genes of the antioxidant system (*Hmox1* 36.2-fold, *Nqo1* 3.6-fold, *Nrf2* 2.4-fold) and the inflammation system (*Cxcl1* 204.1-fold, *Icam1* 14.2-fold, *Il1b* 10.4-fold, *Tlr4* 5.7-fold, *Myd88* 3.4-fold, *Nf-kB1* 2.3-fold, *Nf-kB2* 5.2-fold), cfDNA sensors (*Tlr9* 2.9-fold, *cGas* 4.8-fold, *Sting1* 4.7-fold), and apoptosis/survival signaling (*Bax* 2.7-fold, *Bcl2* 2.9-fold, *Survivin* 3.5-fold, *Tgfb* 5.4-fold).

### 3.2. Dynamics of Inflammation-Related Gene Expression during the First 24 h after cfDNA Treatment

Considering that cfDNA promotes the activation of inflammation in various human and mammalian cells, and no data for brain cells are available, we studied by RT PCR the expression of six proinflammatory genes (*Tlr2*, *Nf-kB1*, *Nf-kB2*, *Myd88*, and *Sting1* and *Nlrp3* (both encoding DNA receptors)) and three genes associated with neuro- and neuritogenesis (*Trkb*, *Bdnf*, *S100a8*, and *S100a9*) at 1, 3, and 24 h in a series of independent experiments. The *Ppia* gene was used as an internal control gene.

**Figure 2:**
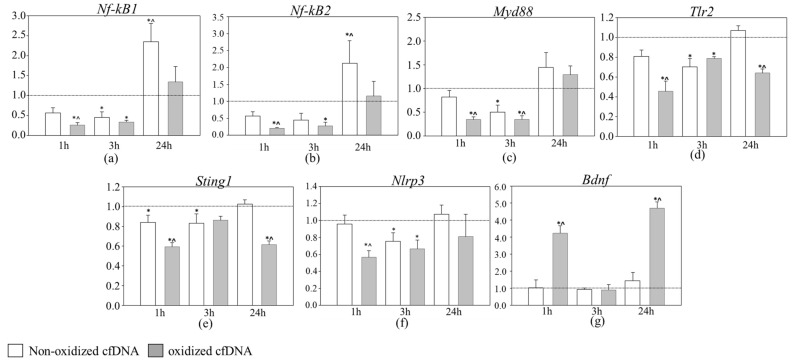
RT PCR gene expression analysis of cells of rat cerebellum after oxidized cfDNA treatment for 1, 3, and 24 h. Bar charts illustrate expression level of (**a**) *Nf-kB1*, (**b**) *Nf-kB2*, (**c**) *Myd88*, (**d**) *Tlr2*, (**e**) *Sting1*, (**f**) *Nlrp3*, and (**g**) *Bdnf*. * *p* < 0.01: non-oxidized cfDNA and oxidized cfDNA vs. control; ^ *p* < 0.01: oxidized cfDNA vs. non-oxidized cfDNA. One-way ANOVA, Holm–Sidak method. The X-axis illustrates experimental conditions: duration from 1 to 24 h and cell exposure to non-oxidized (white) and oxidized (gray) cfDNA. Y-axis illustrates mRNA concentration (mRNA target gene/mRNA *Ppia*) compared to control.

Oxidized cfDNA induced transcription profile changes similar to multiplex analysis were noted after 1–3 h (Figure 2). The results show the dynamics common to all inflammatory genes: the maximum decrease in gene expression was noted after 1 h of incubation (*Nf-kB1* 4.02-fold, *Nf-kB2* 5.29-fold, *Myd88* 3.0-fold, *Nlrp3* 1.92-fold, *Tlr2* 2.3-fold, and *Sting1* 1.7-fold, *p* < 0.0002), followed by a gradual increase by 24 h to the control level (*Nf-kB1*, *Nf-kB2*, *Myd88*, *Nlrp3*) (Figure 2a–c,f). Some genes remained downregulated by the end of one day of incubation (*Tlr2* and *Sting1*, both 1.6-fold, *p* < 0.00001) (Figure 2d,e). The results confirm that *Bdnf* gene expression does not change after 3 h of incubation; however, it is upregulated by oxidized cfDNA at 1 and 24 h (Figure 2g).

Non-oxidized cfDNA exhibited activity later, and it was less prominent compared to the effect of oxidized cfDNA. Therefore, after 3 h of incubation of cells with fragments of non-oxidized cfDNA, a decrease in gene expression was noted for the following inflammation-related genes: *Nf-kB1* (2.4-fold, *p* = 0.004), *Nf-kB2* (2.6-fold *p* > 0.05), and *Myd88* (2.1-fold, *p* = 0.011) (Figure 2). Only after 24 h, in response to non-oxidized cfDNA, was the expression of proinflammatory genes *Nf-kB1* and *Nf-kB2* increased (2.35-fold, *p* = 0.002 and 2.12-fold, *p* = 0.0146, respectively). In contrast, at 24 h and other time points, the oxidized cfDNA had no effect on the expression of inflammation-related genes. We could not assess only the expression of *Trkb*, *S100a8,* and *S100a9* genes in repeated experiments by RT PCR because of weak signals presumably due to low mRNA yield and different sensitivities of the nCounter FLEX Analysis System and RT PCR.

## 4. Discussion

**Figure 3:**
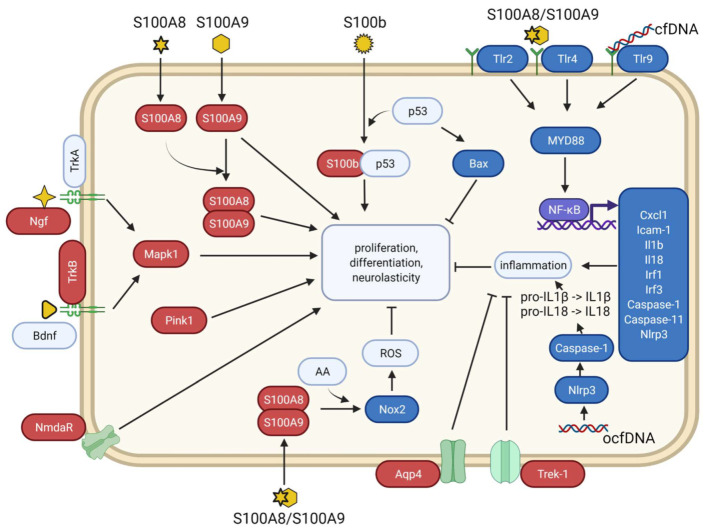
Hypothetical explanation of revealed alterations of gene expression in brain cells after 3 h oxidized cfDNA treatment. Upregulated genes are in red, and downregulated genes are in blue. Genes or cell components whose dynamics were not assessed are in gray, ligands of receptors are in orange, and receptors are in green. AA, arachidonic acid; cfDNA, cell-free DNA; ocfDNA, oxidized cfDNA.

Cell-free DNA is a molecule that transmits stress signals to human and mammalian cells [15]. Oxidized cfDNA molecules or fragments exhibit significantly more activity compared to the non-oxidized form [15,16,17]. There are limited data on the effect of cfDNA on brain cells [16]. In the current work, we expanded the spectrum of the studied genes to assess the signaling cascades that may be involved in the early response of cells to non-oxidized vs. oxidized cfDNA treatment. We focused on the genes of inflammation, oxidation, antioxidation, DNA repair, apoptosis, autophagy, mitophagy, neurogenesis, neuroplasticity, and neuritogenesis. Investigation of no one of these groups was the main aim of our study. The results show that only oxidized cfDNA rapidly (in 3 h) altered the gene expression profile of brain cells. Despite that all upregulated genes (independently of whether pleiotropic they are or not) are involved in multiple functions, one function is common for these genes: they positively control pathways responsible for the development and maturation of new brain cells. These genes regulate neuritogenesis (*S100A8*/*S100A9*) [30], cell proliferation (*S100B*) [31], neurogenesis and neuroplasticity (*TrkB* [32], *Ngf* [33], *NmdaR* [34], *Mapk1* [32,33], *Pink1* [35,36]), and decreased inflammation and are associated with neurogenesis (*Aqp4* [37], *Kcnk2*) [38,39]. On the contrary, genes encoding molecules involved in proinflammatory pathways and diminished neurogenesis (*NF-kB1*, *Myd88*, *Cxcl1*, and others) are suppressed (Figure 3 and Table S1).

An increase in cfDNA concentration occurs under both physiological and pathological conditions [5,6,7,8,9]. We assume that cfDNA has a beneficial or damaging effect depending on the duration of its increased concentration. States such as physical activity are characterized by a brief rise in cfDNA concentration [40], associated with stimulation of neurogenesis in adults [4,41]. Physical activity enhances neurogenesis in the hippocampus and improves cognitive functions by increasing cerebral blood flow, BBB permeability, angiogenesis, and expression of neurotrophic factors [4,41]. In addition, it has been shown that during training, the inflammatory process decreases [42]. It is also known that in humans, the concentrations of nuclear cfDNA and mitochondrial cfDNA (which contains an increased amount of 8-oxo-dG compared to nuclear DNA) are increased for several hours after a treadmill test [40]. In the current work, we show similar changes occurring in brain cells under the action of oxidized cfDNA at the level of gene transcription: an increase in the expression of genes associated with neurogenesis and a decrease in the expression of genes of the inflammatory, oxidation, and apoptosis/survival systems. On the other hand, the long-term increase in cfDNA observed in mental disorders is associated with poor outcomes, apparently due to chronic inflammation [11]. We have previously shown that 11-day exposure to oxidized cfDNA leads to decreased gene expression of the key components of the brain antioxidant system, heme oxygenase 1 (*Hmox1*) and the transcription factor Nrf2 [43]. These results warrant an extended analysis of the brain cell transcriptome following the chronic action of oxidized cfDNA, which has not been performed so far.

There are some limitations in our study. We used rat brain DNA thoroughly purified from proteins. However, in vivo, cfDNA can be formulated within the exosomes [18] or bind to proteins, such as HMGB1 [44]. It has been shown that HMGB1 acts to regulate neurogenesis and neuroplasticity in vivo and in vitro [44]. Whether cfDNA and HMGB1 could act together to target the neurogenesis/neuroplasticity of neural cells requires further studies. There is another limitation of our study: we used cerebellum primary cell culture. Despite data on existence of neurogenesis in adult cerebellum of vertebrates [45], the results may be confirmed in hippocampal cell cultures commonly employed in studies of neurogenesis.

Based on the transcriptional patterns of oxidized cfDNA in brain cells in vitro, as revealed in the present study, we suggest that the biological role of cfDNA might include increasing the preparedness of brain cells for damage. We believe that cfDNA liberated from severely damaged cells warns undamaged cells of possible failure by rapidly activating the transcription of genes that contribute to post-damage recovery of neurons and glial cells.

Thus, oxidized cfDNA is a molecule capable of rapidly switching gene expression toward activation of neurogenesis and neuroplasticity at an early stage of the insult, associated with cell death and liberation of cfDNA. An increased amount of cfDNA in the vicinity of bystander cells may be ruinous for them. Indeed, the chronic enhanced concentration of DNA in plasma is associated with poor outcomes in patients with diseases that involve massive cell death [19,46,47]. These data warrant future studies on determining the optimal non-oxidized cfDNA/oxidized DNA regimen to avoid neurotoxicity and excessive inflammation while maintaining neurogenesis and activating neuroplasticity for neurorepair.

## Figures and Tables

**Table 1:** Primer sequences for RT PCR.

Gene		Sequence	Tm	Length
*Ppia*	F	TCGCGTCTGCTTCGAGCTGT	64.6	135
	R	TGGCACATGAATCCTGGAAT	57.2	
*Nlrp3*	F	GACCAGCCAGAGTGGAATGA	59.38	118
	R	TACAAATCGAGATGCGGGAG	57.49	
*Nf-kB1*	F	GACCGGCAACTCACAGACAG	60.95	170
	R	TCATAGATGGCGTCTGACAC	57.13	
*Nf-kB2*	F	CAATCACCTGCACCAGACAC	59.12	187
	R	TCCACTGTGCAACACTGCCT	62.27	
*Myd88*	F	CTCAGCCTGTCTCCAGGTGT	61.19	148
	R	CAAGACGGGTCCAGAACCAG	60.32	
*Tlr2*	F	GGCTGGAGGTCTCCAGGTCA	63.10	157
	R	AGACCTGGAGCTGCCATCAC	61.91	
*Sting1*	F	GCCATGTCCAGTCCAGGTAC	60.11	153
	R	CAAGATGCCAAGCAAGGCGC	63.15	
*Trkb*	F	AAAGGTTAGAAATCATCAAT	47.66	330
	R	CCAGAGGGGTATTCTTGCTG	57.66	
*Bdnf*	F	CGTCCACGGACAAGGCAACT	62.99	146
	R	CCAGCAGCTCTTCGATCACG	61.14	
*S100a8*	F	CCTCAGTTTGTGCAGAATAA	53.5	191
	R	TATTCTGTAGACATATCCAA	47.8	
*S100a9*	F	GAAGAGGGAGAAAAGAAATG	51.4	179
	R	CTTTGCCGTGGCTGTGGTCA	63.6	

## Supplementary Materials

The following are available online at https://www.mdpi.com/article/10.3390/cimb43030112/s1, Table S1: Multiplex gene expression analysis of cells of rat cerebellum after oxidized and non-oxidized cfDNA treatment for 3 h, Table S2: Preparation of oxidized cell-free DNA from non-oxidized cell-free DNA.
