# Supplementary material for: Oxidized Cell-Free DNA Rapidly Skews the Transcriptional Profile of Brain Cells toward Boosting Neurogenesis and Neuroplasticity
Source: Curr Issues Mol Biol. 2021 Oct 13;43(3):1583–91. doi: 10.3390/cimb43030112 (PMC8929019; doi:10.3390/cimb43030112)
Supplement: Supplementary file 1 [file cimb-43-00112-s001.zip › Supplementary.pdf]

Supplementary

Table S1. Multiplex gene expression analysis in cells of rat cerebellum after oxidized and non-oxidized cfDNA treatment for 3 hours

№	gene	control		ncfDNA		ocfDNA		p adjusted			Power
		M	SD	M	SD	M	SD	ocfDNA/ control	ocfDNA/ ncfDNA	ncfDNA/ control	
Upregulated genes											
1	*S100A9*	1	0,515	1,795	0,665	**43,750**	18,115	<0,0001	<0,0001	>0,05	1
2	*S100b*	1	0,0187	0,911	0,103	**22,974**	8,898	<0,0001	<0,0001	>0,05	1
3	*TrkB*	1	0,0434	0,990	0,0700	**7,363**	3,210	<0,0001	<0,0001	>0,05	1
4	*S100A8*	1	0,209	0,976	0,486	**5,232**	2,237	<0,0001	<0,0001	>0,05	1
5	*AQP4*	1	0,0453	0,965	0,134	**4,023**	2,453	<0,0001	<0,0001	>0,05	1
6	*Pink1*	1	0,0185	0,957	0,0640	**3,861**	1,972	<0,0001	<0,0001	>0,05	1
7	*Ngf*	1	0,128	0,787	0,170	**3,394**	1,724	<0,0001	<0,0001	>0,05	1
8	*Kcnk2*	1	0,0234	1,012	0,0915	**3,237**	1,445	<0,0001	<0,0001	>0,05	1
9	*Mapk1*	1	0,0437	0,991	0,0573	**2,876**	1,307	<0,0001	<0,0001	>0,05	1
10	*Nmdar*	1	0,0430	0,955	0,0690	**2,688**	1,454	<0,0001	<0,0001	>0,05	1
Downregulated genes											
1	*Cxcl1*	1	0,0188	0,855	0,102	**204,104**	67,349	<0,0001	<0,0001	>0,05	1
2	*NOX2*	1	0,0222	0,953	0,0620	**44,796**	32,477	<0,0001	<0,0001	>0,05	1
3	*Hmox1*	1	0,0749	0,995	0,0983	**36,199**	17,343	<0,0001	<0,0001	>0,05	1
4	*Igf-1*	1	0,0338	1,008	0,0759	**31,785**	19,867	<0,0001	<0,0001	>0,05	1
5	*Tlr2*	1	0,0467	0,940	0,0719	**19,805**	7,608	<0,0001	<0,0001	>0,05	1
6	*Icam-1*	1	0,0635	0,944	0,146	**14,177**	4,932	<0,0001	<0,0001	>0,05	1
7	*Il1b*	1	0,0498	0,731	0,221	**10,367**	4,063	<0,0001	<0,0001	>0,05	1
8	*CD14*	1	0,0277	0,839	0,170	**9,722**	3,876	<0,0001	<0,0001	>0,05	1
9	*Il18*	1	0,0745	0,997	0,0849	**7,977**	4,268	<0,0001	<0,0001	>0,05	1
10	*Tlr4*	1	0,0356	1,015	0,101	**5,744**	2,528	<0,0001	<0,0001	>0,05	1
11	*CD36*	1	0,108	1,254	0,0956	**5,602**	1,939	<0,0001	<0,0001	>0,05	1
12	*Tgfb*	1	0,0227	1,047	0,0537	**5,547**	2,129	<0,0001	<0,0001	>0,05	1
13	*Nfkb2*	1	0,0713	0,981	0,0894	**5,168**	3,054	<0,0001	<0,0001	>0,05	1
14	*Mb21d1 (cGAS)*	1	0,245	1,230	0,190	**4,832**	1,857	<0,0001	<0,0001	>0,05	1

Table S1. (continuation)

№	gene	control		ncfDNA		ocfDNA		p_{adjusted}			Power
		M	SD	M	SD	M	SD	ocfDNA/ control	ocfDNA/ ncfDNA	ncfDNA/ control	
						Downregulated genes					
15	*Tmem173 (STING)*	1	0,0281	0,977	0,0876	**4,747**	1,999	<0,0001	<0,0001	>0,05	1
16	*Wnt5a*	1	0,164	0,951	0,0537	**4,502**	2,740	<0,0001	<0,0001	>0,05	1
17	*Aim2*	1	0,0668	1,138	0,175	**4,285**	2,737	<0,0001	0,000149	>0,05	1
18	*Brca1*	1	0,0839	1,151	0,292	**4,133**	2,376	<0,0001	<0,0001	>0,05	1
19	*Nqo1*	1	0,0212	1,022	0,0544	**3,616**	1,473	<0,0001	<0,0001	>0,05	1
20	*Nlrp3*	1	0,0369	0,947	0,236	**3,591**	2,190	0,0001	<0,0001	>0,05	1
21	*Survivin*	1	0,0410	0,978	0,123	**3,498**	1,666	<0,0001	<0,0001	>0,05	1
22	*Irf3*	1	0,119	1,075	0,0625	**2,482**	0,865	<0,0001	<0,0001	>0,05	1
23	*Irf1*	1	0,152	1,232	0,0987	**3,418**	1,303	<0,0001	<0,0001	>0,05	1
24	*Myd88*	1	0,0376	1,099	0,0884	**3,416**	1,383	<0,0001	<0,0001	>0,05	1
25	*Caspase-1*	1	0,117	0,976	0,113	**3,138**	1,326	<0,0001	<0,0001	>0,05	1
26	*Bcl2*	1	0,0735	1,011	0,116	**2,927**	1,044	<0,0001	<0,0001	>0,05	1
27	*tlr9*	1	0,139	0,942	0,249	**2,910**	1,014	<0,0001	<0,0001	>0,05	1
28	*OLR1*	1	0,254	1,276	0,231	**2,841**	1,196	<0,0001	<0,0001	>0,05	1
29	*Bax*	1	0,0552	1,029	0,0665	**2,687**	0,929	<0,0001	<0,0001	>0,05	1
30	*Ngfr*	1	0,132	0,965	0,103	**2,595**	0,901	<0,0001	<0,0001	>0,05	1
31	*Nrf2*	1	0,0399	0,967	0,0498	**2,422**	0,955	<0,0001	<0,0001	>0,05	1
32	*Nfkb1*	1	0,0306	0,938	0,0519	**2,287**	0,818	<0,0001	<0,0001	>0,05	1
33	*Plcg1*	1	0,0231	1,037	0,0554	**2,253**	0,791	<0,0001	<0,0001	>0,05	1
34	*Creb*	1	0,150	0,988	0,0502	**2,252**	0,859	<0,0001	<0,0001	>0,05	1
35	*Gsdmd*	1	0,0307	1,154	0,128	**2,239**	0,812	<0,0001	<0,0001	>0,05	1
36	*Hif1a*	1	0,0630	0,957	0,0924	**2,108**	0,736	<0,0001	<0,0001	>0,05	1
37	*caspase-11*	1	0,0181	0,982	0,0491	**2,089**	0,724	<0,0001	<0,0001	>0,05	1

Note: One-way ANOVA, Holm-Sidak method. p_{adj} <0,0001 (all shown genes). Numbers in bold indicate statistical significance.
Abbreviations: ocfDNA - oxidized cfDNA, ncfDNA – non-oxidized cfDNA.
